# Association between early ondansetron administration and in-hospital mortality in critically ill patients: analysis of the MIMIC-IV database

**DOI:** 10.1186/s12967-022-03401-y

**Published:** 2022-05-14

**Authors:** Yingying Fang, Chao Xiong, Xinghe Wang

**Affiliations:** 1 Department of Phase I Clinical Trial Center, Beijing Shijitan Hospital, Capital Medical University, Beijing, 100038 China; 2 Department of Anaesthesiology, Fuwai Hospital, National Centre for Cardiovascular Diseases, Chinese Academy of Medical Sciences and Peking Union Medical College, Beijing, 100037 China

**Keywords:** Ondansetron, Antiemetic, Intensive care unit, Mortality

## Abstract

**Background:**

While ondansetron (OND) is widespread availability, the contribution of OND to improve patient outcomes among intensive care unit (ICU) patients has not been examined. This study aimed to illustrate the association between early OND use and in-hospital mortality in critically ill patients and investigate whether this association differed according to OND dose.

**Methods:**

The MIMIC-IV database was employed to identify patients who had and had not received OND. Statistical approaches included multivariate logistic regression, propensity score matching (PSM), and propensity score-based inverse probability of treatment weighting (IPTW) models to ensure the robustness of our findings.

**Results:**

In total, 51,342 ICU patients were included. A significant benefit in terms of in-hospital mortality was observed in the OND patients compared to the non-OND group in the early stage [odds ratio (OR) = 0.75, 95% CI 0.63–0.89, p < 0.001]. In the circulatory system group, the early OND administration was associated with improved in-hospital mortality in ICU patients (OR 0.48, 95% CI 0.34–0.66; P < 0.001). The risk of in-hospital mortality was also lower in early OND users than in non-OND users both in the medical admission group and the surgical ICU admission group, and ORs were 0.57 (95% CI 0.42–0.76; P < 0.001) and 0.79 (95% CI 0.62–0.91; P < 0.001), respectively.

A positive role of daily low- and moderate-dose OND treatment in early-stage was showed on the in-hospital mortality in PSM cohort, and the ORs were 0.75 (95% CI 0.62–0.90; P < 0.001) and 0.63 (95% CI 0.43–0.91; P < 0.001), respectively. The relationship between the daily low- and moderate-dose of OND and in-hospital mortality was also significant in ICU patients with cardiovascular diseases, and ORs were 0.51(95% CI 0.36–0.73; P < 0.001), and 0.26(95% CI 0.11–0.65; P < 0.001), respectively. Daily low-to-moderate dose of OND was also associated with in-hospital mortality in ICU entire cohort.

**Conclusions:**

Early OND use is closely associated with lower in-hospital mortality in ICU patients. Daily low-to-moderate dose of OND application is protective against in-hospital mortality. This association is more evident in the circulatory system group.

**Graphical Abstract:**

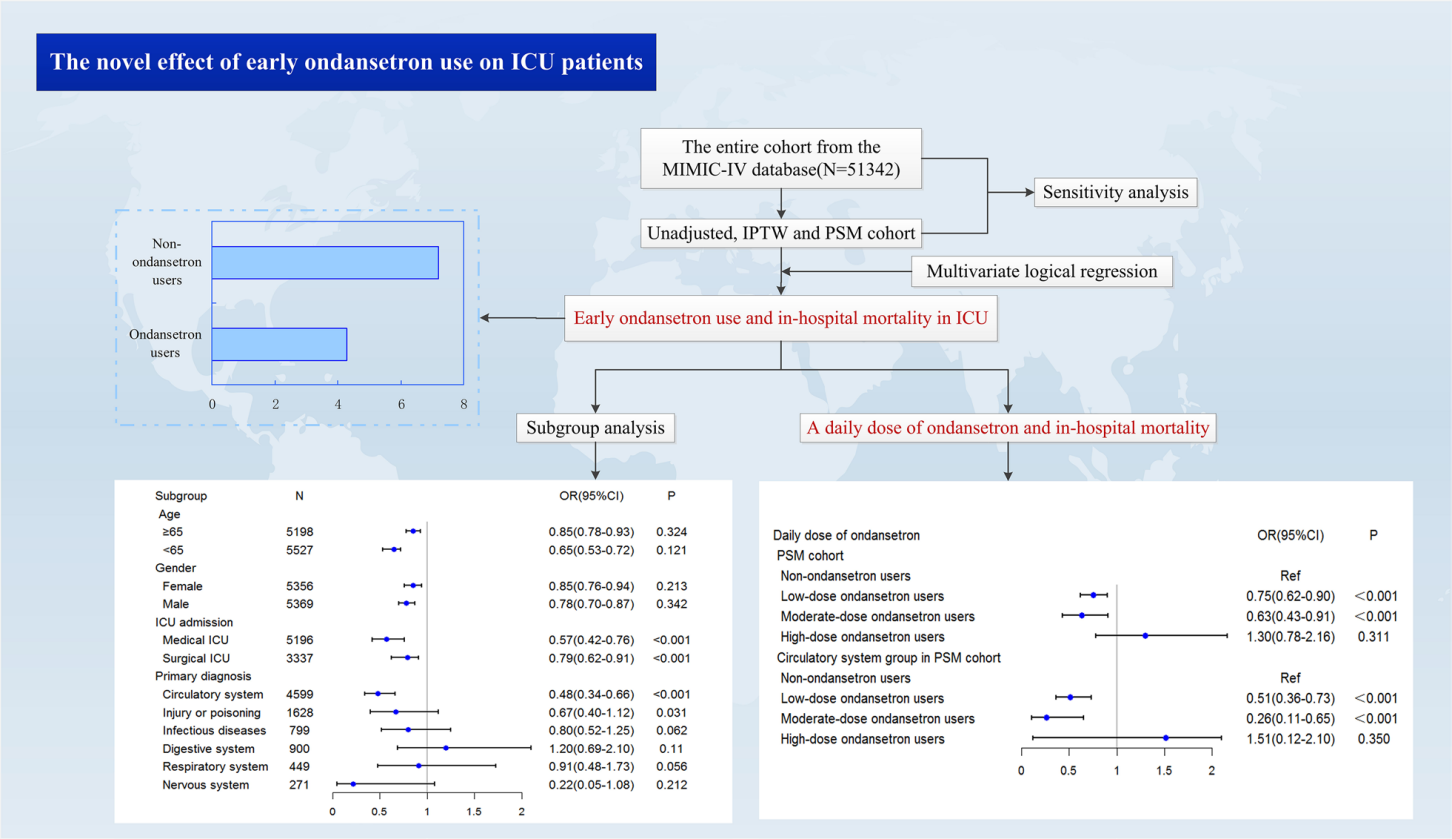

**Supplementary Information:**

The online version contains supplementary material available at 10.1186/s12967-022-03401-y.

## Background

Ondansetron (OND) is the earliest used serotonin 5-hydroxytryptamine (5-HT_3_) receptor antagonist as an antiemetic drug with widespread applications. Early use of OND would effectively prevent and alleviate nausea and vomiting for critically ill patients to reduce complications and the mortality rate in ICU [1–3]. Interestingly, the latest study found that OND could be used to decrease mortality in the coronavirus disease 2019(COVID-19) inpatients [4]. In addition, OND has been illustrated the potentially pleiotropic effect, including neuroprotection [5], renal protection [6, 7], and anticoagulation [8, 9]. However, research on the survival benefit of initial use of OND, particularly in the critically ill, is lacking. Therefore, this study aimed to evaluate the association between early treatment of OND and in-hospital mortality in ICU patients and whether this association differed according to OND dose.

## Methods

### Database

We enrolled a cohort of patients admitted into ICU, treated with and without OND, from a real-world and publicly available clinical database named Medical Information Mart for Intensive Care Database IV (MIMIC-IV version 1.0), and maintained by Beth Israel Deaconess Medical Center in Boston, MA, USA from 2012 to 2019. We were permitted to extract data from the database, and all reporting followed the Strengthening the Reporting of Observational Studies in Epidemiology (STROBE) guidelines.

### Study population

The medical records of all adult patients aged at least 18 years admitted to ICU were analyzed. We chose the first ICU admission for patients who were enrolled into the ICU more than once. Those who discharged or died within 48 h after ICU admission was excluded. Patients who were encountered with missing variable data (medication information) and outcome data (in-hospital mortality) were removed.

### Data extraction

Data collected included (1) demographic characteristics [sex, age (yr), ethnicity]; (2) the admission type; (3) Sequential Organ Failure Assessment (SOFA) score, Simplified Acute Physiology Score II (SAPS II) score; (4) comorbidities (myocardial infarct, congestive heart failure, peripheral vascular disease, cerebrovascular disease, dementia, chronic pulmonary disease, rheumatic disease, peptic ulcer disease, liver disease, paraplegia, renal disease, diabetes, malignant cancer, metastatic solid tumour, and acquired immune deficiency syndrome (AIDS)); (5) treatment measures (vasopressors, mechanical ventilation, and renal replacement therapy).

The data were obtained from MIMIC-IV using Structured Query Language (SQL) with pgAdmin (version 4). The Simplified Acute Physiology Score (SAPS) II and Sequential Organ Failure Assessment (SOFA) scores were calculated within the first 24 h after ICU admission. Early application of OND referred to the OND application from 24 h before ICU admission to 48 h after ICU admission (− 24 h to 48 h). A daily dose of early OND application referred to the average dose of 3 days (− 24 h to 48 h). Treatment measures were collected on the first day admitted to ICU.

### Main exposure and study endpoints

Low-dose OND was defined as > 0 mg per day and ≤ 8 mg per day. Moderate-dose OND was defined as > 8 mg per day and ≤ 16 mg per day. High-dose OND was defined as > 16 mg per day. The endpoint of this study was in-hospital mortality.

### Statistical analysis

Continuous variables were expressed as the median and interquartile range (IQR) because of their non-normal distribution. Categorical variables were described as the number and percentage (%). Two-group comparisons (with OND vs. without OND group) were conducted with Manne Whitney U test or Chisquared test as appropriate.

Multivariate logistic regression analysis was conducted to assess the association between early ondansetron use and outcomes, with the results expressed as odds ratios (ORs) and corresponding 95% confidence intervals (95% CIs). The mortality outcomes adjusting for confounding variables, shown in Table 1, were selected based on p-value < 0.05 in univariate analysis and potential confounders decided by previous studies and clinical expertise.

**Table 1:** Baseline characteristics between groups before and after PSM

Variables	Entire cohort (n = 51,342)		SMD	PSM cohort (n = 10,724)		SMD
	Non-ondansetron (n = 45,980)	Ondansetron (n = 5362)		Non-ondansetron (n = 5362)	Ondansetron (n = 5362)	
Age [median (IQR)]	64.7 (52.2,76.5)	59.6 (46.5,70.2)	0.165	64.1 (51.5,75.9)	64.6 (53.0,74.2)	0.006
Men, n (%)	26,146 (56.9)	2670 (49.8)	0.142	2699 (50.3)	2670 (49.8)	0.016
Ethnicity, n (%)			0.054			0.023
Black	4352 (9.5)	402 (7.5)		493 (9.2)	402 (7.5)	
White	31,023 (67.5)	3635 (67.8)		3451 (64.4)	3635 (67.8)	
Hispanic	1598 (3.5)	190 (3.5)		215 (4.0)	190 (3.5)	
Others	3604 (7.8)	449 (8.4)		477(8.9)	449 (8.5)	
Admission type, n (%)			0.201			0.006
Medical/non-surgical	31,901 (69.4)	2794 (52.1)		3253 (60.7)	2794 (52.1)	
Elective surgical	6678 (14.5)	1840 (34.3)		925 (17.3)	1840 (34.3)	
Non-elective surgical	7401 (16.1)	728 (13.6)		1184 (22.1)	728 (13.6)	
Comorbidities at ICU admission, n (%)						
Myocardial infarct	7307 (15.9)	872 (16.3)	0.010	863 (16.1)	872 (16.3)	0.023
Congestive heart failure	11,196 (24.3)	970 (18.1)	0.154	930 (17.3)	970 (18.1)	0.002
Peripheral vascular disease	5067 (11.0)	549 (10.2)	0.025	547 (10.2)	549 (10.2)	0.017
Cerebrovascular disease	7249 (15.8)	853 (15.9)	0.004	870 (16.2)	853 (15.9)	0.011
Dementia	1715 (3.7)	109 (2.0)	0.102	100 (1.9)	109 (2.0)	0.025
Chronic pulmonary disease	10,987 (23.9)	1044 (19.5)	0.108	1045 (19.5)	1044 (19.5)	0.006
Rheumatic disease	1487 (3.2)	173 (3.2)	0.001	148 (2.8)	173 (3.2)	0.001
Peptic ulcer disease	1302 (2.8)	121 (2.3)	0.037	108 (2.0)	121 (2.3)	0.023
Liver disease	6741 (14.6)	651 (12.1)	0.070	637(19.9)	651 (12.1)	0.002
Diabetes with CC	13,764 (29.9)	16,178 (30.1)	0.097	1600 (29.8)	1618 (30.1)	0.009
Paraplegia	2431 (5.3)	220 (4.1)	0.056	227 (4.2)	220 (4.1)	0.016
Renal disease	8207 (17.8)	786 (14.7)	0.087	724 (13.5)	786 (14.7)	0.014
Malignant cancer	5826 (12.7)	603 (11.2)	0.044	614 (11.5)	603 (11.2)	0.002
Metastatic solid tumor	2802 (6.1)	304 (5.7)	0.018	325 (6.1)	304 (5.7)	0.011
AIDS	264 (0.6)	15 (0.3)	0.045	23 (0.4)	15 (0.3)	0.007
Need of support in the first 24 h, n (%)						
Vasopressors	12,606 (27.4)	1735 (32.4)	0.108	1764 (32.9)	1735 (32.4)	0.005
MV	12,044 (26.2)	1499 (28.0)	0.040	1504 (28.0)	1499 (28.0)	0.001
RRT	877 (1.9)	93 (1.7)	0.013	90 (1.7)	93 (1.7)	0.008
ICU types, n (%)			0.122			0.009
CCU	5539 (12.0)	302 (5.6)		588 (11.0)	302 (5.6)	
SICU	7307 (15.9)	603 (11.2)		915 (17.1)	603 (11.2)	
NSICU	1006 (2.2)	298 (5.6)		114 (2.1)	298 (5.6)	
CVICU	7628 (16.6)	1744 (32.5)		1102 (20.6)	1744 (32.5)	
TSICU	6111 (13.3)	616 (11.5)		791 (14.8)	616 (11.5)	
MICU	9140 (19.9)	595 (11.1)		865 (16.1)	595 (11.1)	
MICU/SICU	7779 (16.9)	738 (13.8)		816 (15.2)	738 (13.8)	
Primary diagnosis, n (%)			0.051			0.007
Circulatory system	16,820 (36.6)	2469 (46.0)		2130 (39.7)	2469 (46.0)	
Injury or poisoning	7416 (16.1)	648 (12.1)		980 (18.3)	648 (12.1)	
Infectious diseases	4532 (9.9)	347 (6.5)		452 (8.4)	347 (6.5)	
Digestive system	4247 (9.2)	471 (8.8)		429 (8.0)	471 (8.8)	
Respiratory system	3183 (6.9)	149 (2.8)		300 (5.6)	149 (2.8)	
Immunity diseases	1288 (2.8)	165 (3.1)		151 (2.8)	165 (3.1)	
Nervous system	1166 (2.5)	150 (2.8)		121 (2.3)	150 (2.8)	
Genitourinary system	743 (1.6)	65 (1.2)		46 (0.9)	65 (1.2)	
Severity of illness						
SOFA score [median (IQR)]	4 (3,10)	4 (3,9)	0.144	4 (3,9)	4.(3,10)	
SAPS II score [median (IQR)]	33 (13,50)	32 (12,48)	0.051	32 (12,48)	32 (12,49)	
Outcome						
In-hospital mortality, n (%)	3481 (7.6)	229 (4.3)		301 (5.6)	229 (4.3)	

PSM: propensity acore matching; ICU: intensive care unit; AIDS: acquired immune deficiency syndrome; MV: mechanical ventilation; RRT: renal replacement therapy; CCU: coronary care unit; SICU: surgical intensive care unit; NSICU: neuro surgical intensive care unit; CVICU: cardiac vascular intensive care unit; TSICU: trauma surgical intensive care unit; MICU: medical intensive care unit; IQR: interquartile range; SOFA: sequential organ failure assessment; SAPS II: Simplified Acute Physiology Score II

Propensity score matching (PSM) and propensity score-based inverse probability of treatment weighting (IPTW) were utilized to ensure the robustness of our findings [10, 11]. Logistic regression analysis was carried out in the cohort to perform OR assessment in in-hospital mortality between early OND users and non-OND users, and the confounding variables included (1) demographic characteristics [sex, age(yr), ethnicity]; (2) the admission type(admission type, the type of icu care unit); (3) primary diagnosis; (4) SOFA within the first 24 h after ICU admission, SAPSii score within the first 24 h after ICU admission; (5) comorbidities (myocardial infarct, congestive heart failure, peripheral vascular disease, cerebrovascular disease, dementia, chronic pulmonary disease, rheumatic disease, peptic ulcer disease, liver disease, paraplegia, renal disease, diabetes, malignant cancer, metastatic solid tumour, and acute respiratory distress syndrome(AIDS)); (6) treatment measures (vasopressors, mechanical ventilation, and renal replacement therapy).

The results were described as ORs with 95% CIs. In the PSM model, one-to-one nearest neighbour matching with a calliper width of 0.1 was applied in our study. For the IPTW model, a pseudo-population was generated according to the propensity score. Standardized mean differences (SMDs) were computed to evaluate the efficiency of an unadjusted cohort, PSM and IPTW. Notably, baseline profiles were well balanced between the two groups with SMDs that were less than 5% for all variables (Additional file 1: Fig. S1).

As for the PSM cohort, subgroup analysis was conducted to explore whether the association between early OND administration and in-hospital mortality was modified by age, sex, ICU admission and primary diagnosis. Primary diagnosis was classified into the circulatory system, injury or poisoning, infectious diseases, digestive system, respiratory system and nervous system.

The relationship between the daily dose of OND and in-hospital mortality was also evaluated by multivariable logistic regression analysis in the entire population, PSM cohort and circulatory system group after PSM, and the confounding variables included age, gender, SOFA score and SAPS II score. Statistical analysis was performed using R 3.5.3 software for windows and Python 3.7.3. A p-value < 0.05 was considered statistically significant.

## Results

### Population and baseline characteristics

**Fig. 1:**
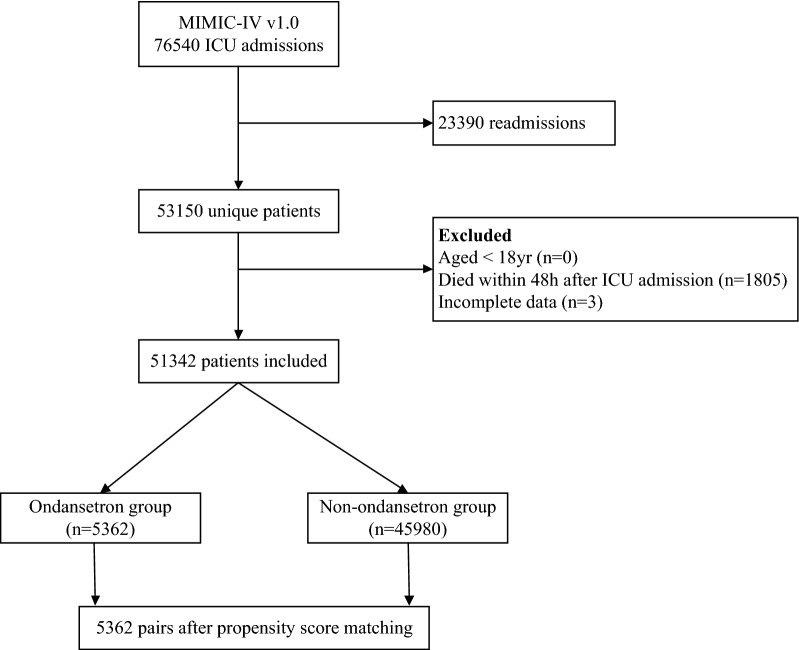
Flowchart of patient selection for the study. MIMIC-IV: Medical Information Mart for Intensive Care Database IV; ICU: intensive care unit

During the study period, 53,150 critically ill patients were enrolled in ICU. Excluding the patients according to the exclusion criteria, we identified 51,342 eligible ICU individuals and collected the data of clinical risk variables and outcome variables. An additional table file shows the missing data in the variables in more detail (see Additional file 2: Table S1]. Of these, 5362 used OND in the early stage (11.66%) during their ICU stay and 45,980 patients did not receive early ondansetron treatment (88.34%). After PSM, 5362 early OND users and 5362 non-OND users were included in the final analysis (Fig. 1).

As shown in Table 1, there were meaningful differences in gender, admission types between the early OND group and the non-OND group both in the whole cohort and in the PSM cohort. Early OND treatment was more common in men. The proportion of patients with early OND treatment was larger during elective surgical admission. The incidence of early OND use was considerably higher in cardiovascular surgical ICU (CVICU). Patients with disorders of the circulatory system in ICU were more likely to be given OND in the early period.

### Relationship between early OND use and in-hospital mortality

The overall in-hospital mortality was 7.2% (3710/51342). The in-hospital mortality of the OND group was 4.3% (229/5362), compared with 7.6% (3481/45980) for the non-OND group in Table 1.

**Fig. 2:**
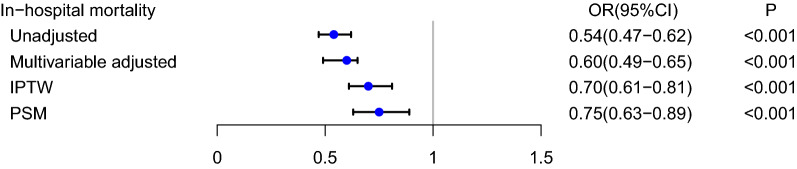
Association between ondansetron use and in-hospital morality of ICU patients. OR: odds ratio; CI: confidence interval; ICU: intensive care unit; Unadjusted: without adjustment; Multivariable adjusted: adjusted for all the baseline variables shown in Table 1. PSM: propensity score matching. IPTW: inverse probability of treatment weighting

Compared with patients who were not administered OND, patients who received early OND were associated with a 46% decrease in the risk of in-hospital mortality in the unadjusted model (OR: 0.54, 95% CI 0.47–0.62, p < 0.001). After adjusting for confounding factors, the OR for early OND administration in the multivariate logistic regression was 0.60 (95% CI 0.49–0.65, p < 0.001). The results of the IPTW (OR: 0.70, 95% CI 0.61–0.81, p < 0.001) and PSM (OR: 0.75, 95% CI 0.63–0.89, p < 0.001) models demonstrated a significant beneficial effect of early OND use on in-hospital mortality among ICU patients (Fig. 2).

### Subgroup analysis

**Fig. 3:**
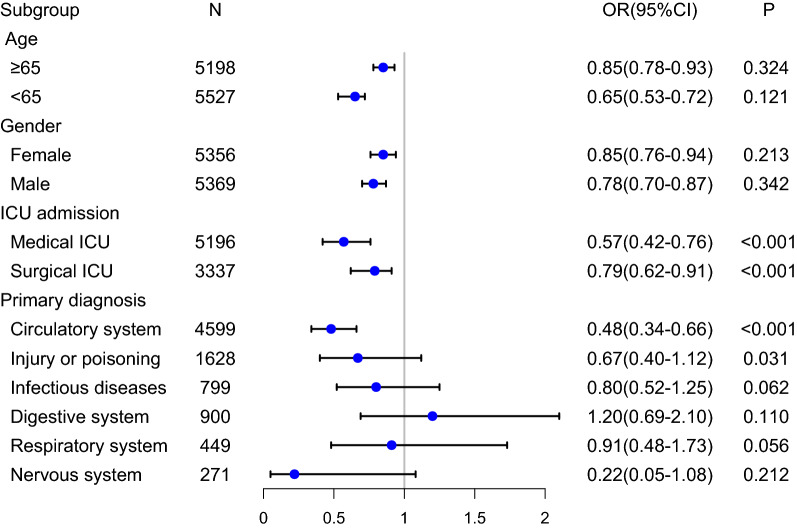
The association between ondansetron administration and in-hospital mortality in subgroup. OR: odds ratio, CI: confidence interval

The number of patients in each subgroup was shown in Fig. 3. In the circulatory system, early OND use was associated with decreasing in-hospital mortality in ICU patients (OR 0.48, 95% CI 0.34–0.66; p < 0.001). The improved outcome was also observed in the medical and the surgical ICU group, and the ORs were 0.57 (95% CI 0.42–0.76; P < 0.001), and 0.79 (95% CI 0.62–0.91; P < 0.001), respectively.

### A daily dose of OND and in-hospital mortality

In the PSM cohort, we found that low- and moderate-dose OND treatment was associated with a reduced risk of in-hospital mortality when compared with the non-OND group, and the ORs were 0.75 (95% CI 0.62–0.90; p < 0.001) and 0.63 (95% CI 0.43–0.91; p < 0.001), respectively. There was not a distinguishing difference in the risk of in-hospital mortality in patients who received early high-dose OND treatment (OR 1.30; 95% CI 0.78–2.16; p = 0.311). In circulatory system group, a remarkable beneficial impact of daily low- and moderate-dose OND treatment on in-hospital mortality was also found, and OR of 0.51 (95% CI 0.37–0.73; P < 0.001), OR of 0.26 (95% CI 0.11–0.65; P < 0.001), respectively (Table2).

**Table 2:** Multivariate logistic analysis in the entire and PSM cohort, and circulatory system group

Daily dose of OND	Entire cohort (N = 51,342)	P	PSM cohort (10,724)	P	Circulatory system group in PSM cohort (n = 4599)	P
	OR (95% CI)		OR (95% CI)		OR (95% CI)	
Nn-OND useors	Ref		Ref		Ref	
Low-dose OND users	0.70 (0.60–0.83)	< 0.001	0.75 (0.62–0.90)	< 0.001	0.51 (0.36–0.73)	< 0.001
Moderate-dose OND users	0.65 (0.45–0.94)	< 0.001	0.63 (0.43–0.91)	< 0.001	0.26 (0.11–0.65)	< 0.001
High-dose OND users	1.58 (0.96–2.61)	0.074	1.30 (0.78–2.16)	0.311	1.51 (0.12–2.10)	0.350

In the entire cohort, the risk of in-hospital mortality was 30% lower in early low-dose OND users than non-OND users (OR 0.70, 95% CI 0.60–0.83; P < 0.001), and 35% decrease in moderate-dose OND users than non-OND users (OR 0.65, 95% CI 0.35–0.94; P < 0.001) (Table 2).

## Discussion

This study showed that early OND use was significantly associated with lower in-hospital mortality in ICU patients, and there was probably a remarkable association between daily low to moderate dose of OND and in-hospital mortality. Early OND usage would be strongly connected to in-hospital mortality in critically ill patients with cardiovascular diseases.

Similar to our study, the present study demonstrated that OND may promote mortality in critically ill patients. First, a latest study found that OND use would reduce 30-day all-cause mortality in COVID-19 inpatients [4]. Recent studies explained that 5-HT_3_ receptor antagonists could prevent the rotavirus-induced release of serotonin (5-HT) from human enterochromaffin cells and activates brain structures involved in nausea and vomiting [12, 13]. A similar study illustrated that the impaired 5-HT-dependent signalling would delay the intracellular transport of incoming virions by altering the distribution of early endosomes in disassembly kinetics, resulting in decreased infectivity and impaired cell killing by diverse viruses including reovirus, chikungunya virus (CHIKV), mouse hepatitis virus (MHV) and two unrelated RNA viruses [14, 15]. In addition, OND would be neuroprotective. A recent animal study indicated that OND penetrates into the central nervous system (CNS), which may depend on the expression level of efflux transporters at the capillaries of the blood–brain barrier (BBB), such as P-glycoprotein (Pgp) encoded by the ABCB1 gene [16]. OND treatment significantly attenuated BBB breakdown, edema formation, glial fibrillary acidic protein (GFAP) and heat shock protein (HSP 72 kDa) expression and neuronal injuries [5]. Importantly, potentially favourable effects of OND on reduced in-hospital mortality in acute kidney injury (AKI) patients in the ICU were reported depending on the MIMIC-III database, the eICU database and the MIMIC-IV database. Based on the comparison of gene expression signatures, the latest study illustrated that the advantageous effect of OND might be elicited through the NF-KB pathway and JAK-STAT pathway [6, 7]. A recent pharmacoepidemiology study reported that OND was also associated with a significant decrease in 90-day mortality on the High-Density Intensive Care (HiDenIC-15) database containing intensive care data for 13 hospitals across Western Pennsylvania [17]. Last but not least, OND would inhibit agonist-induced platelet aggregation. And the negative effect of OND on platelet aggregation might manifest through the attenuation of agonist-induced IP3 production and MAPK phosphorylation resulting in suppressed intracellular Ca (2+) mobilization, TXB2 formation, and ATP release [8]. Thus, OND would become a promising drug for improving prognosis in critically ill patient with common conditions in the ICU.

The results of the subgroup analysis are also notable in this study. The relationship between the worse outcome and early OND use was more evident in ICU patients suffering from cardiovascular diseases. This might be the result of the following mechanisms. Importantly, OND therapy exerts its lower effect on in-hospital mortality may involve the prevention of cardiac inhibitory and the improvement of hemodynamics and cardiovascular collapse [18–20]. Additionally, the effect of OND therapy on reducing mortality could differ according to age or comorbidities.

In the medical ICU population, the association between early OND use and the outcome was more remarkable than in the surgical ICU population. For one thing, patients in the medical ICU have relatively more severe illnesses than patients in the surgical ICU, where the main purpose of early OND application is to prevent postoperative nausea and vomiting (PONV). In contrast with antiemetic prophylaxis, the early OND administration in the majority of patients is to relieve nausea and vomiting caused by critical illness in the medical ICU. For another thing, the pleiotropic effect of OND on in-hospital mortality could differ according to diverse disease types, comorbidities as well as different sample sizes.

In our study, the daily low- and moderate-dose of OND were probably related to the reduced in-hospital mortality in ICU patients. On the one hand, the latest research mentioned that there was no significant difference among diverse doses of OND to reduce emesis. A recent study established a dose–response relationship for OND in patients and showed that a single 8 mg OND was equivalent to a single 32 mg OND in terms of the antiemetic effect [21]. Some researchers hypothesized there could be a plateau in the therapeutic efficacy of OND which further dose escalation does not improve outcome rather than potently increasing the adverse cardiovascular events [21, 22]. On the other hand, the FDA has issued multiple safety announcements regarding potential risks for dysrhythmias secondary to QTc prolongation following application of OND at doses higher than that which are typically utilized in the ICU. In response, drug manufacturers currently recommended that a daily dose of OND should not be more than 16 mg [23]. The dose recommendation (< 16 mg per day) is compatible with the range of low- and moderate-OND doses in our study. Last but not least, the risk of significant QTc prolongation and arrhythmia is more remarkable in ICU patients receiving multiple medications, especially in poor metabolizers when combined with other medications which are metabolized through the cytochrome P450 (CYP) 2D6 pathway [23]. Thus, further experiments in vivo and in vitro are necessary to confirm the optimal daily dose of OND in the early stage in ICU patients.

Our study had several limitations. First, given its retrospective observational design, many confounders needed to be controlled by PSM, IPTW or multivariable adjustment. Second, although we performed PSM to adjust for confounders in this study, there might be unmeasured confounders and selection bias. For example, the severity of disease was insufficiently measured or included in the PSM and thus might affect OND use in this study. Moreover, patients who applied with OND, despite having similar comorbidities after PSM, might be a different quality of outpatient care or other macro-level health care. Therefore, adherence to OND use in OND users was also unclear during hospitalization among patients in this study. Finally, this was a single-centre study. The results need to be validated by multicenter trials.

## Conclusions

Early OND use is significantly associated with lower in-hospital mortality among critically ill patients. Daily low-to-moderate OND dose is valuable related to in-hospital mortality in ICU. This association might be greater in those with cardiovascular diseases. Our results may be beneficial for the rational use of OND in critically ill patients, especially with cardiovascular diseases.

## Supplementary Information

**Additional file 1: Fig. S1.** Standardized mean differences (SMDs) of variables in unmatched, propensity score matching (PSM) and inverse probability of treatment weighting (IPTW) models. cc: complications; vent 1st day: mechanical ventilation within the first 24 h after ICU admission; vaso 1st day: vasopressors within the first 24 h after ICU admission; sofa 1st day: sequential organ failure assessment score within the first 24 h after ICU admission; sapsii 1st day: simplified acute physiology score ii score within the first 24 h after ICU admission; Unmatched: unmatched data(red line); Matched: propensity score matching (PSM) (green line); Weighted: inverse probability of treatment weighting (IPTW) (blue line).

**Additional file 2: Table S1.** Percentage of missing data in the variables in the interest.

## Data Availability

The datasets used in our study are available from the first author and corresponding authors on reasonable request.

## Abbreviations

OND: Ondansetron
PSM: Propensity score matching
IPTW: Propensity score-based inverse probability of treatment weighting
5-HT_3_: 5-Hydroxytryptamine
STROBE: Strengthening the reporting of observational studies in epidemiology
AIDS: Acquired immune deficiency syndrome
SQL: Structured query language
CI: Confidence interval
OR: Odds ratio
ICU: Intensive care unit
IQR: Interquartile range
MIMIC-IV: Multiparameter Intelligent Monitoring in Intensive Care Database IV
MV: Mechanical ventilation
RRT: Renal replacement therapy
SOFA: Sequential Organ Failure Assessment
SAPS II: Simplified Acute Physiology score II
SMD: Standardized mean difference
CCU: Coronary care unit
SICU: Surgical intensive care unit
NSICU: Neuro surgical intensive care unit
CVICU: Cardiac vascular intensive care unit
TSICU: Trauma surgical intensive care unit
MICU: Medical intensive care unit
COVID-19: Coronavirus disease 2019
CHIKV: Chikungunya virus
MHV: Mouse hepatitis virus
PONV: Postoperative nausea and vomiting
AKI: Acute kidney injury
