# Supplementary figures and images for: Association between early ondansetron administration and in-hospital mortality in critically ill patients: analysis of the MIMIC-IV database
Source: J Transl Med. 2022 May 14;20:223. doi: 10.1186/s12967-022-03401-y (PMC9107069; doi:10.1186/s12967-022-03401-y)

variable

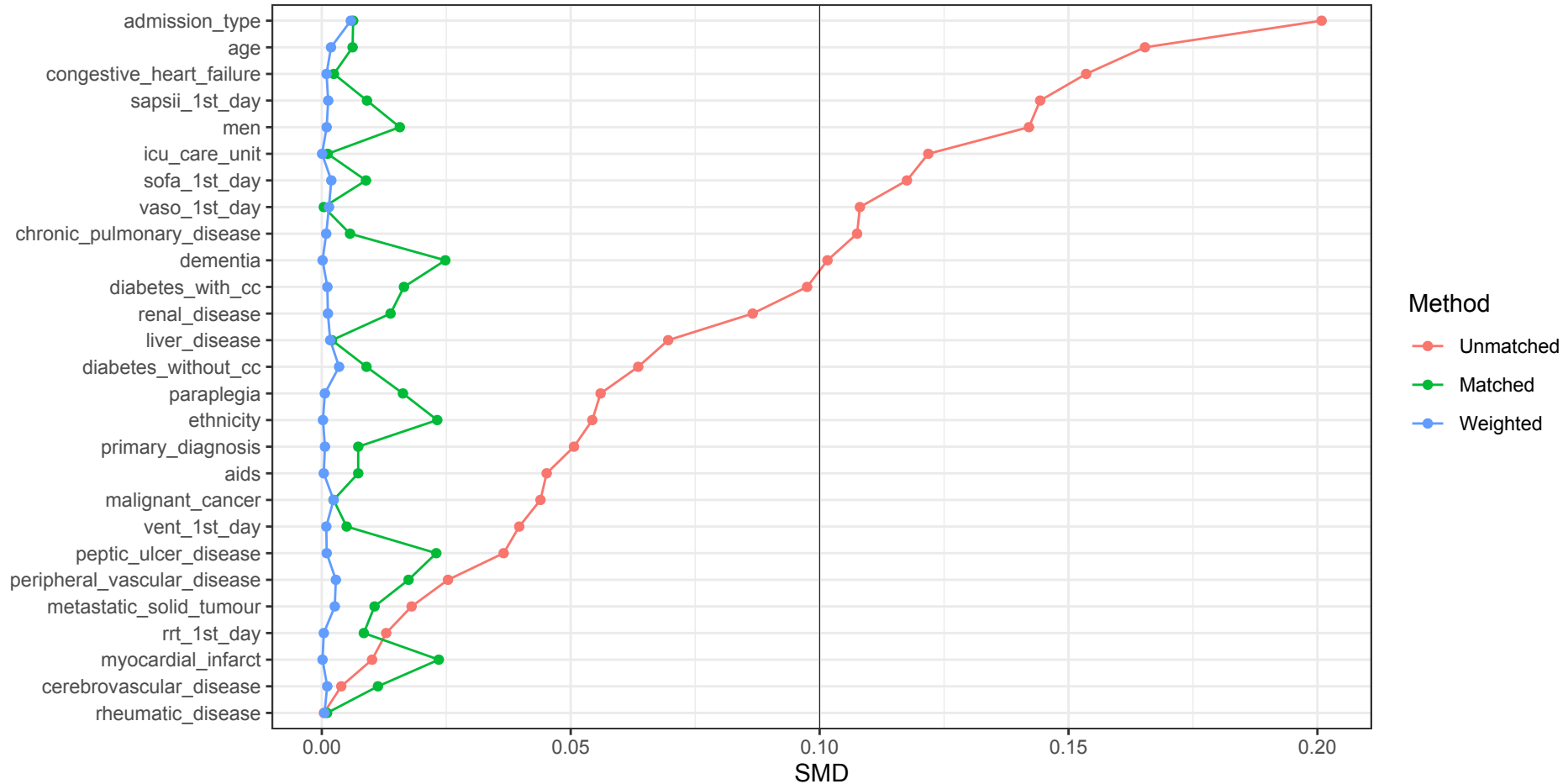

Supplement: Supplementary file 1 — Additional file 1: Fig. S1. Standardized mean differences (SMDs) of variables in unmatched, propensity score matching (PSM) and inverse probability of treatment weighting (IPTW) models. cc: complications; vent 1st day: mechanical ventilation within the first 24 h after ICU admission; vaso 1st day: vasopressors within the first 24 h after ICU admission; sofa 1st day: sequential organ failure assessment score within the first 24 h after ICU admission; sapsii 1st day: simplified acute physiology score ii score within the first 24 h after ICU admission; Unmatched: unmatched data(red line); Matched: propensity score matching (PSM) (green line); Weighted: inverse probability of treatment weighting (IPTW) (blue line). [file 12967_2022_3401_MOESM1_ESM.pdf]
